# Otitis in Patients With Community-Acquired Bacterial Meningitis: A Nationwide Prospective Cohort Study

**DOI:** 10.1093/cid/ciae221

**Published:** 2024-04-24

**Authors:** Martina Ranzenigo, Thijs M van Soest, Erik F Hensen, Paola Cinque, Antonella Castagna, Matthijs C Brouwer, Diederik van de Beek

**Affiliations:** Department of Neurology, Amsterdam Neuroscience, Amsterdam UMC, University of Amsterdam, Meibergdreef, Amsterdam, The Netherlands; Unit of Infectious Diseases, IRCCS San Raffaele Scientific Institute, Milan, Italy; Unit of Infectious Diseases, Vita-Salute San Raffaele University, Milan, Italy; Department of Neurology, Amsterdam Neuroscience, Amsterdam UMC, University of Amsterdam, Meibergdreef, Amsterdam, The Netherlands; Department of Otorhinolaryngology and Head and Neck Surgery, LUMC, Leiden, The Netherlands; Unit of Infectious Diseases, IRCCS San Raffaele Scientific Institute, Milan, Italy; Unit of Infectious Diseases, IRCCS San Raffaele Scientific Institute, Milan, Italy; Unit of Infectious Diseases, Vita-Salute San Raffaele University, Milan, Italy; Department of Neurology, Amsterdam Neuroscience, Amsterdam UMC, University of Amsterdam, Meibergdreef, Amsterdam, The Netherlands; Department of Neurology, Amsterdam Neuroscience, Amsterdam UMC, University of Amsterdam, Meibergdreef, Amsterdam, The Netherlands

**Keywords:** otitis, community-acquired bacterial meningitis, otogenic meningitis, otology, ear-nose-throat surgery

## Abstract

**Background:**

Otitis is commonly associated with community-acquired bacterial meningitis, but the role of ear surgery as treatment is debated. In this study, we investigated the impact of otitis and ear surgery on outcome of adults with community-acquired bacterial meningitis.

**Methods:**

We analyzed episodes of adults with community-acquired bacterial meningitis from a nationwide prospective cohort study in the Netherlands, between March 2006 and July 2021.

**Results:**

A total of 2548 episodes of community-acquired bacterial meningitis were evaluated. Otitis was present in 696 episodes (27%). In these patients the primary causative pathogen was *Streptococcus pneumoniae* (615 of 696 [88%]), followed by *Streptococcus pyogenes* (5%) and *Haemophilus influenzae* (4%). In 519 of 632 otitis episodes (82%) an ear-nose-throat specialist was consulted, and surgery was performed in 287 of 519 (55%). The types of surgery performed were myringotomy with ventilation tube insertion in 110 of 287 episodes (38%), mastoidectomy in 103 of 287 (36%), and myringotomy alone in 74 of 287 (26%). Unfavorable outcome occurred in 210 of 696 episodes (30%) and in 65 of 696 episodes was fatal (9%). Otitis was associated with a favorable outcome in a multivariable analysis (odds ratio 0.74; 95% confidence interval [CI] .59–.92; *P* = .008). There was no association between outcome and ear surgery.

**Conclusions:**

Otitis is a common focus of infection in community-acquired bacterial meningitis in adults, with *S. pneumoniae* being the most common causative pathogen. Presence of otitis is associated with a favorable outcome. Ear surgery's impact on the outcome of otogenic meningitis patients remains uncertain.

## Teaser

In adults, community-acquired bacterial meningitis often involves otitis, with the leading pathogen being *Streptococcus pneumoniae*. The presence of otitis is associated with a favorable outcome, however the impact of ear surgery on the outcome remains unclear.

Community-acquired bacterial meningitis is associated with high mortality and morbidity and is most commonly caused by *Streptococcus pneumoniae* [1]. In patients presenting with bacterial meningitis, distant foci of infection occur often and may include otitis [2, 3], sinusitis [4], pneumonia [5], or endocarditis [6]. Otitis has been described to occur in approximately 20% of patients presenting with community-acquired bacterial meningitis [7–9]. Otogenic meningitis, that is, meningitis with concomitant otitis, can be caused by local spread of the pathogen through infected bony structures or by hematogenous spread [10]. In a prospective cohort study involving 696 adults with community-acquired bacterial meningitis, otitis or sinusitis was identified as an independent prognostic factor for unfavorable outcome (odds ratio [OR] 1.80 [95% confidence interval {CI} 1.13–2.84]) [11], an association that may be explained by the association between otitis and pneumococcal meningitis, although other studies recognized a protective role of otitis on outcome [7, 9]. It remains unclear whether patients with bacterial meningitis and otitis benefit from surgery aimed at resolving the ear infection. Studies on this topic are few, and there are no evidence-based guidelines [12–15]. Myringotomy with or without ventilation tube insertion and different types of mastoidectomy are common ear-nose-throat (ENT) surgeries performed in these patients [12–15]. In this study, we investigated the characteristics of patients with otogenic bacterial meningitis and the impact of otitis and ear surgery on their outcome.

## METHODS

The MeninGene study is a nationwide prospective cohort study on community-acquired bacterial meningitis in the Netherlands [7, 16, 17]. Patients aged 16 years or older were identified following a daily update by the Netherlands Reference Laboratory for Bacterial Meningitis (NRLBM), which receives approximately 90% of the cerebrospinal fluid (CSF) isolates of patients with bacterial meningitis [18, 19]. The daily update included the names of hospitals and treating physicians. Subsequently, treating physicians were informed about the study by telephone. Patients or legal representatives received written information about the study and were asked to give written informed consent. Treating physicians could also contact the researchers 24/7 to include a patient in the study. Data were prospectively collected through an online case record form (CRF), including clinical characteristics, laboratory results, microbiological results, clinical course, treatment, and outcome [18, 19].

We included all patients with a proven bacterial meningitis, defined either as a positive CSF culture, positive CSF polymerase chain reaction (PCR), positive CSF antigen test, or the combination of a positive blood culture with at least 1 CSF finding predictive of bacterial meningitis, according to the criteria defined by Spanos and colleagues (CSF leucocyte count >2000 cells/mm^3^, polymorphonuclear leucocyte count >1180 cells/mm^3^, glucose level <1.9 mmol/L, or CSF/ blood glucose ratio <0.23) [20]. We excluded patients with hospital-acquired meningitis, with a neurosurgical device and those who had undergone neurosurgery or had significant head trauma within 1 month prior to hospital presentation. The presence or absence of otitis on admission is a standard question in the case record form and the diagnosis was made on clinical and/or radiological findings. We divided our sample into 2 groups based on the presence or absence of otitis at admission. In otogenic meningitis episodes, the discharge letters were screened in order to collect data on ENT consultancy and surgery. Myringotomy alone (defined as an incision in the eardrum without the insertion of a ventilation tube), myringotomy with ventilation tube insertion and mastoidectomy (defined as any surgical procedure comprising the exenteration of the air cells of the mastoid process of the temporal bone) were considered surgical treatments targeted at resolving the otitis. ENT consultancy was requested at the discretion of the attending physician. To evaluate whether the hospital type influenced decisions on ENT surgical treatments, hospitals were divided in 3 groups: academic hospitals, large non-academic teaching hospitals, and small non-academic hospitals. At hospital discharge, all patients underwent a neurological examination and outcome was scored using the Glasgow Outcome Scale, ranging from 1 (death) to 5 (no or mild neurological deficit). A favorable outcome was defined as a score of 5 and unfavorable outcome as a score of 1–4. Data on hearing impairment were collected at discharge in surviving patients and were defined by the treating physician.

Group differences were tested with the Fisher's exact test for categorical and ordinal variables and the Mann-Whitney *U* test for continuous variables. Univariable and multivariable logistic regression was used to examine the association between potential predictors and the likelihood of an unfavorable outcome. Potential predictors were selected based on pathophysiological interest and earlier research [7, 11]. Linearity was assessed visually, and variables were categorized if no linearity was found. We used multiple imputation for missing data in the multivariable analysis. We used all predictors together to impute missing values using the Mice package (version 3.13.0). We combined the coefficients of 60 rounds of imputation to obtain the final estimates for the multivariable model. ORs and 95% CIs were used to quantify the strength of these associations. *P* values <.05 were considered statistically significant. Analyses were conducted in R (version 4.0.3).

## RESULTS

From 1 March 2006 to 1 July 2021, 2548 episodes of community-acquired bacterial meningitis in 2503 patients were included. The median age was 61 (interquartile range [IQR] 49–70) years, and 1291 out of 2548 (51%) episodes occurred in male patients. On admission, otitis was diagnosed in 696 episodes (27%) in 690 patients. In 119 of these 696 episodes (17%) of otogenic meningitis a coexisting diagnosis of sinusitis was made (Table 1). Median age of patients with otogenic meningitis was 60 (IQR 49–68) years and 332 of 696 episodes (48%) occurred in male patients. Immunocompromising predisposing conditions were present in 158 of 696 episodes (23%), most commonly diabetes mellitus (102 of 689 [15%]) and immunosuppressive therapy (34 of 689 [5%]). Upon admission, an altered mental status (defined as a Glasgow Coma Scale [GCS] below 14) was observed in 517 of 683 episodes (76%), whereas in 170 of 683 episodes (25%) the patient was comatose (defined as Glasgow Coma Scale <8). The classic triad of fever, altered mental status, and neck stiffness was reported in 318 of 642 episodes (50%).

**Table 1.** Characteristics at Admission, Complications, Treatment, and Outcome of Episodes With Otogenic Meningitis on Admission

Patients Characteristics	Episodes With Otogenic Meningitis n = 696	Patients Characteristics	Episodes With Otogenic Meningitis n = 696
Age	60 (49–68)	**Pathogens**	
Male sex	332/696 (48%)	Positive blood culture	467/603 (77%)
Pretreatment with antibiotics	113/672 (17%)	Positive CSF culture	653/696 (94%)
Symptoms <24 h	361/681 (53%)	*Streptococcus pneumoniae*	615/696 (88%)
**Predisposing conditions**		*Neisseria meningitidis*	7/696 (1%)
Sinusitis	119/696 (17%)	*Haemophilus influenzae*	28/696 (4%)
Pneumonia	28/682 (4%)	*Listeria monocytogenes*	2/696 (0%)
Endocarditis	1/682 (0%)	*Staphylococcus aureus*	0/696 (0%)
Alcoholism	20/691 (3%)	*Streptococcus pyogenes*	38/696 (5%)
Active Cancer	15/695 (2%)	Other	6/696 (1%)
Diabetes mellitus	102/689 (15%)	**Treatment**	
Immunosuppressive therapy	34/689 (5%)	Dexamethasone	633/681 (93%)
**Signs and symptoms on admission**		ENT consultancy yes	519/632^a^ (82%)
Altered mental state (GCS < 14)	517/683 (76%)	Ear surgery^b^	287/519 (55%)
GCS < 8	170/683 (25%)	Myringotomy alone	74/287 (26%)
Fever (>38 C)	547/674 (81%)	Myringotomy with ventilation tube insertion	110/287 (38%)
Heart rate (beats/min)^c^	100 (85–115)	Mastoidectomy	103/287 (36%)
Systolic blood pressure (mmHg)^d^	147 (130–169)	**Intensive care unit admission**	235/604 (39%)
Diastolic blood pressure (mmHg)^e^	80 (70–90)	**Systemic complications**	179/679 (26%)
Meningitis triad^f^	318/642 (50%)	Respiratory failure	131/663 (20%)
Neck stiffness	481/633 (76%)	Circulatory shock	49/657 (7%)
**Imaging on admission**		Pneumonia	67/648 (10%)
Normal Brain CT/MRI	232/696 (33%)	**Neurological complications**	155/681 (23%)
Generalized edema	69/634 (11%)	Venous sinus thrombosis	22/639 (3%)
Subdural empyema	7/628 (1%)	Seizures	96/668 (14%)
Brain abscess	3/628 (0%)	Brain abscess	11/374 (3%)
Mastoid opacification	420/620 (68%)	Subdural empyema	18/372 (5%)
Sinus opacification	160/595 (27%)	Cerebrovascular accident	52/650 (8%)
**Blood indexes of inflammation**		**Outcome**	
Leukocyte count (10^9^/L)^g^	18 (13–23)	Hearing impairment	306/558 (54%)
C-reactive protein (mg/L)^h^	164 (84–278)	GOS	
**CSF indices of inflammation**		1 (death)	65/696 (9%)
Leukocytes (cell/mm^3^)^i^	3610 (1324–7656)	2 (vegetative state)	0/696 (0%)
Protein (g/L)^j^	4.1 (2.4–6.4)	3 (severely disabled)	27/696 (4%)
		4 (moderately disabled)	118/696 (17%)
		5 (mild or no disability)	486/696 (70%)

Data are number/number evaluated (%) and continuous values are median (Q1–Q3) unless otherwise stated.

Abbreviations: CSF, cerebrospinal fluid infection; CT, computerized tomography; ENT, ear-nose-throat; GOS, Glasgow Outcome Score; MRI, magnetic resonance imaging.

^a^Age was known in 656 episodes.

^b^632 episodes with otogenic meningitis had data available on ENT consultancy/ear surgery.

^c^Heart rate was known in 656 episodes.

^d^Systolic blood pressure was known in 665 episodes.

^e^Diastolic blood pressure was known in 665 episodes.

^f^Triad of fever, neck stiffness, and change in mental status.

^g^Blood leucocyte count was known in 687 episodes.

^h^C-reactive protein was known in in 673 episodes.

^i^CSF leucocyte count was known in 667 episodes.

^j^CSF protein was known in 651 episodes.

A lumbar puncture was performed in all episodes. CSF examination revealed a median white cell count of 3610 (IQR 1324–7656) cells/mm^3^. At least 1 specific CSF finding predictive of bacterial meningitis was present in 618 of 696 episodes (89%). Cranial imaging was performed on admission for all the 696 episodes and abnormalities were recorded in 464 cases (67%), most commonly mastoid opacification (in 420 of 620 episodes [68%]) and sinus opacification (in 160 of 595 [27%]). Subdural empyema and brain abscesses were identified at admission in 7 (1%) and 3 (0.5%) of 628 episodes, respectively. Positive blood cultures were present in 467 of 603 (77%) episodes, whereas the CSF cultures revealed the causative pathogen in 653 of 696 episodes (94%). The most common causative pathogens were *Streptococcus pneumoniae* (615 of 696 [88%]), *Streptococcus pyogenes* (38 of 696 [5%]), and *Haemophilus influenzae* (28 of 696 [4%]).

**Figure 1.**
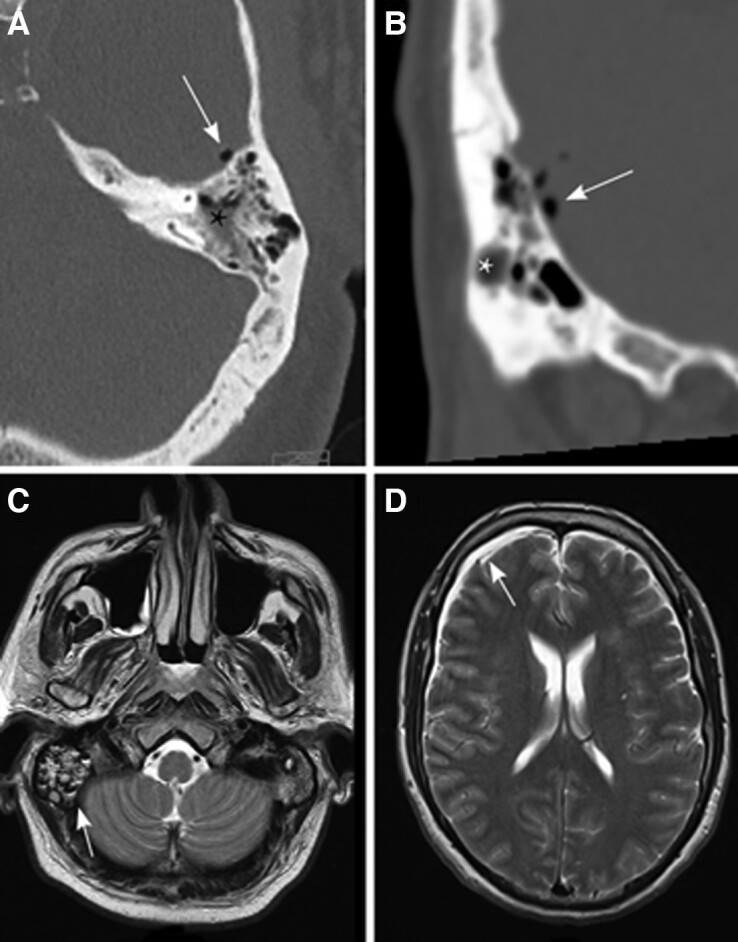
Cranial imaging of patients with otogenic meningitis. Axial (*A*) and coronal (*B*) computed tomography showing mastoid opacification (asterisk) and intracranial air (arrow). Axial T2 weighed cranial MRI showing opacification of the right mastoid in a meningitis patients (*C*, arrow), and hyperintense subdural fluid collection consistent with empyema (*D*). Abbreviation: MRI, magnetic resonance imaging.

Initial antibiotic treatment consisted of a third-generation cephalosporin and amoxicillin or ampicillin in 343 of 672 episodes (51%), monotherapy with a third-generation cephalosporin in 183 (27%), monotherapy with amoxicillin, ampicillin, or penicillin in 90 (13%), and other antibiotic regimens in 56 (8%). Adjunctive dexamethasone was administered in 633 (93%) of 681 episodes. Systemic complications occurred in 179 of 679 episodes (26%), with respiratory failure being the most common (131 of 663 episodes, 20%). Intensive care unit admission was necessary for 235 of 604 episodes (39%). Neurological complications occurred in 155 of 681 episodes (23%), most commonly seizures (96 of 668 [14%]), cerebrovascular infarction (52 of 650 [8%]), and subdural empyema (18 of 372 [5%]; no data available in other episodes), Figure 1. Cerebral venous sinus thrombosis developed in 22 (3%) of 639 episodes. Neurological deficit onset presented in 12 of 18 episodes (66%) with subdural empyema and in 14 of 22 (64%) with cerebral venous sinus thrombosis.

Data on surgical treatment were available in 632 of 696 episodes (91%). An ENT specialist was consulted in 519 of 632 episodes (82%); in 287 episodes (55%) a surgical procedure was performed targeting the otitis. The most common type of surgery was myringotomy with ventilation tube insertion (110 of 287 [38%]), followed by mastoidectomy (103 of 287 [36%]) and myringotomy alone (74 of 287 [26%]; Table 1). In 27 episodes of mastoidectomy, this latter procedure was preceded by myringotomy with ventilation tube insertion, while in 7 episodes by myringotomy alone. The rate of surgical intervention was similar between academic and non-academic hospitals although mastoidectomy was performed more frequently in academic hospitals (30 of 50 [60%]; Supplementary Table 1), compared to large non-academic teaching hospitals (33 of 130 [25%]; *P* < .001) or to small non-academic hospitals (40 of 107 [37%]; *P* = .012).

The case fatality rate was 65 of 696 (9%). Unfavorable outcome occurred in 210 of 696 episodes with otogenic meningitis (30%). Hearing impairment at discharge was present in 306 of 558 (54%) episodes who survived with data available. No differences in hearing loss and case fatality rate were seen among episodes of otogenic meningitis that did not undergo any surgery, compared to the episodes where surgery was performed. Episodes in which mastoidectomy was performed had a longer length of stay (18 days [IQR 12–32] vs 15 days [IQR 11–19]; *P* < .001) compared to episodes with no surgery, Table 2. Also, a multivariable analysis was performed in otogenic meningitis episodes, to assess the association between type of surgery and outcome. No association was demonstrated (Supplementary Table 2).

**Table 2.** Differences Among Episodes of Otogenic Meningitis Where No Surgery Was Performed and Episodes That Underwent Surgery

	No Surgery (n = 345)	Myringotomy Alone (n = 74)	Myringotomy With Ventilation Tube Insertion (n = 110)	Mastoidectomy (n = 103)
Unfavorable outcome	106/345 (31%)	19/74 (26%)	25/110 (23%)	40/103 (39%)
Mortality	35/345 (10%)	6/74 (8%)	6/110 (5%)	9/103 (9%)
Days to discharge	15 (11–19)	14 (11–16)	14 (11–18)	18 (12–32)***
Hearing loss	142/286 (50%)	39/62 (63%)	55/94 (59%)	46/82 (56%)

No surgery group was compared with each of the other 3 groups, singularly.

*** *P* value <.001.

In a multivariable analysis including all 2548 episodes of bacterial meningitis (Table 3), presence of otitis at admission, was independently associated with a favorable outcome (OR 0.74 [0.59–0.92]; *P* = .008). The predictive effect of otitis remained robust when including *S. pneumoniae* in the analysis (OR 0.78 [0.62–0.98]; *P* = .003; Supplementary Table 3).

**Table 3.** Factors Associated With Unfavorable Outcome in Patients With Community-Acquired Bacterial Meningitis

Characteristics	Favorable Outcome (n = 1554)	Unfavorable Outcome (n = 979)	Univariable Odds Ratio for Unfavorable Outcome (95% CI)	Multivariable Odds Ratio for Unfavorable Outcome (95% CI)	*P* Value of Multivariable Analysis
Age, years^a^	58 (43–67)	66 (56–75)	1.04 (1.03–1.05)	1.03 (1.02–1.04)	<.001
Sex, male	771/1554 (50%)	514/979 (53%)	1.12 (.96–1.32)	1.22 (1.00–1.48)	.044
Pretreatment with antibiotics	164/1509 (11%)	79/941 (8%)	0.76 (.58–1.00)	0.84 (.60–1.16)	.28
Symptoms <24 h	764/1516 (50%)	388/909 (43%)	0.74 (.63–.87)	0.73 (.59–.89)	.003
Immunocompromised*	383/1521 (25%)	373/948 (39%)	1.92 (1.61–2.28)	1.36 (1.11–1.67)	.004
Otitis	486/1554 (31%)	210/979 (21%)	0.60 (.50–.72)	0.74 (.59–.92)	.008
Pneumonia	104/1512 (7%)	130/906 (14%)	2.53 (1.95–3.29)	1.17 (.85–1.61)	.39
Headache	1217/1434 (85%)	509/722 (70%)	0.39 (.32–.47)	0.73 (.59–.92)	.002
Nausea	831/1339 (62%)	366/714 (51%)	0.63 (.53–.74)	0.95 (.77–1.18)	.65
Rash	149/1405 (11%)	52/851 (6%)	0.58 (.43–.79)	0.85 (.56–1.29)	.44
Neck stiffness	1088/1438 (76%)	582/860 (68%)	0.68 (.57–.81)	0.73 (.59–.91)	.006
Cranial nerve palsy	73/1375 (5%)	96/767 (13%)	2.44 (1.84–3.24)	2.29 (1.61–3.25)	<.001
Focal neurologic deficit	262/1440 (18%)	230/805 (29%)	1.77 (1.47–2.13)	1.33 (1.05–1.69)	.018
Heart rate (beats per min)^b^	96 (81–109)	103 (88–120)	1.20 (1.16–1.24)	1.15 (1.09–1.20)	<.001
Fever >38°C	1134/1516 (75%)	645/926 (70%)	0.77 (.45–.92)	0.71 (.57–.89)	.004
Score on Glasgow Coma Scale^c^	12 (10–14)	10 (8–13)	0.85 (.83–.87)	0.88 (.85–.91)	<.001
Positive blood culture	980/1330 (74%)	674/829 (81%)	1.67 (1.38–2.04)	1.10 (.85–1.42)	.46
C-reactive protein (mg/L)	154 (68–260)	234 (120–348)			
<50	281/1507 (19%)	92/926 (10%)	Reference	Reference	
50–150	457/1507 (30%)	202/926 (22%)	1.32 (1.00–1.76)	1.24 (.90–1.71)	.19
>150	769/1507 (51%)	632/926 (68%)	2.51 (1.95–3.24)	1.75 (1.29–2.39)	<.001
Thrombocyte count	207 (159–258)	180 (129–245)			
<150	314/1478 (21%)	300/889 (34%)	1.97 (1.64–2.36)	1.27 (1.02–1.58)	0.033
150–450	1134/1478 (77%)	568/889 (64%)	Reference	Reference	
>450	30/1478 (2%)	21/889 (2%)	1.38 (.79–2.36)	1.00 (.51–1.95)	>0.99
CSF protein (g/L)	3.5 (2.0–5.8)	4.5 (2.6–6.8)			
<0.5	36/1463 (2%)	19/903 (2%)	Reference	Reference	
0.5–1.5	206/1463 (14%)	76/903 (8%)	0.67 (.37–1.24)	.81 (.38–1.70)	.57
>1.50	1221/1463 (83%)	808/903 (89%)	1.22 (.71–2.16)	0.88 (.42–1.84)	.74
CSF white cell count (cell per µL)	3060 (1020–7645)	1252 (215–4829)			
<100	90/1481 (6%)	157/920 (17%)	3.90 (2.96–5.15)	2.53 (1.76–3.63)	<.001
100–999	273/1481 (18%)	271/920 (29%)	2.23 (1.83–2.73)	1.99 (1.56–2.53)	<.001
1000–10 000	855/1481 (58%)	389/920 (42%)	Reference	Reference	
>10 000	263/1481 (18%)	103/920 (11%)	0.86 (.67–1.10)	0.79 (.59–1.06)	.11
CSF:blood glucose ratio	0.1 (0.01–0.31)	0.02 (0.01–0.17)			
<0.25	922/1414 (65%)	664/824 (81%)	1.83 (1.29–2.64)	2.12 (1.25–3.61)	.006
0.25–0.5	394/1414 (28%)	126/824 (15%)	0.85 (.58–1.26)	1.17 (.68–2.02)	.56
>0.5	98/1414 (7%)	34/824 (4%)	Reference	Reference	

The study included 2533 out of 2548 episodes of community-acquired meningitis; 15 episodes did not have data on outcome. Data are median (interquartile range [IQR]) or n/N (%), unless stated otherwise. The multivariable analysis used an imputed dataset with 60 imputation rounds, all variables in the table were entered in the multivariable logistic regression model simultaneously. Abbreviations: CI, confidence interval; CSF, cerebrospinal fluid.

*Patient was defined immunocompromised if either one of the following conditions were present: active cancer, immunosuppressive therapy, history of alcohol abuse, diabetes mellitus, splenectomy, human immunodeficiency virus (HIV) infection.

^a^Evaluated in 2533 episodes.

^b^Evaluated in 2395 episodes; odds ratio is for an increase of ten beats per min.

^c^Evaluated in 2490 episodes; odds ratio is for a one point increase.

## DISCUSSION

Our study showed that otitis is common in adults with community-acquired bacterial meningitis, occurring in 27% of the cases. The most common causative pathogen in otogenic meningitis was *S. pneumoniae,* isolated in 88% of the cases, aligning with previous studies [21–24]. Other pathogens that occur relatively frequently in otogenic meningitis are *Streptococcus pyogenes* and *Haemophilus influenzae.* Otitis was found to be associated with a favorable outcome in patients with community-acquired bacterial meningitis. In a substantial proportion of patients, ENT specialists were consulted and ear surgery was undertaken in approximately half of these patients. However, our observational study did not demonstrate a significant association between surgery and outcome.

In patients with otogenic meningitis, abnormalities were frequently observed on cranial imaging. High rates of mastoid and sinus opacification were detected consistent with ENT infection but also relatively high rates of other severe complications of bacterial meningitis, such as subdural empyema and sinus thrombosis [13, 14, 25–27]. Our previous study showed that subdural empyema occurs in 3% of adults with community-acquired bacterial meningitis, with 75% of these cases being associated with concomitant otitis or sinusitis [28]. Although sinus thrombosis complicating bacterial meningitis is also rare (1%), a previous study indicated that 67% of these patients had concomitant otitis or sinusitis [29]. Therefore, cranial imaging should be performed in all patients with otogenic meningitis, with repeated imaging if focal neurological deficits or seizures occur during clinical course.

We found a case fatality rate of 9%, consistent with previous literature [22, 30, 31]. In our multivariable analysis, otitis was associated with a favorable outcome, aligning with previous research [7]. This favorable outcome may be attributed due to local spread of the infection, as opposed to the hematogenous spread in other cases of bacterial meningitis [32]. This is also supported by the lower rate of systemic complications in episodes with otogenic meningitis. Although the similar rate of positive blood culture between episodes with or without otogenic meningitis would suggest similar rates of hematogenous spread, it has been demonstrated that bacteremia occurs also secondary to meningitis following direct spread from otitis or sinusitis [33]. Nevertheless, although speculative, an alternative hypothesis could be that patients with otogenic meningitis or the family of these patients might have a lower threshold to seek medical attention.

In our cohort, an ENT surgeon was consulted in most patients and ear surgery targeted at resolving the causative ear infection was undertaken in approximately half of these patients. The most common surgical procedure was myringotomy with ventilation tube insertion, followed by mastoidectomy and myringotomy alone. Mastoidectomy was more likely to be performed if patients presented to a tertiary care facility. Mastoidectomy is a more complex surgical procedure, whereas myringotomy with or without ventilation tube insertion is a less complicated procedure that can be performed under local anesthesia. We did not find an association between surgery and outcome, nor surgery type and outcome, however these results must be interpreted with caution because of confounders of indications to surgery. Selection bias may influence outcomes as episodes elected for surgery may have been in worse condition before surgery or more resistant to initial antibiotic therapy, whereas episodes that responded well to initial treatment may have been less likely to undergo surgery. If this is the case, it would help explain the longer hospitalization of surgical patients. Furthermore, the observation that patients that undergo surgery have a comparable outcome to patients that do not, may be viewed as an argument for the judicious selection of patients for (the different types of) surgery. Unfortunately, there is no consensus on indications, timing and preferred type of surgery in otogenic meningitis patients, with varying recommendations ranging from immediate surgery [12, 26] to a more conservative approach based on the response to antimicrobial therapy [14, 15, 27].

There are several limitations to this study. First, we only included patients who underwent lumbar puncture, potentially introducing selection bias leading to underestimation of the mortality rate. Second, diagnosis of otitis at admission was based on either imaging or a clinical diagnosis rather than ENT evaluation. This did not allow us to distinguish between type of ear diseases (*ie*, acute otitis media, chronic otitis media, chronic otitis media with cholesteatoma), or define cases of recurrent otitis. Furthermore, we lacked information about the timing of surgery and indication for surgery, which could be crucial in determine the impact of surgery on outcome. Finally, in episodes with multiple sources of infection upon presentation, it is not clear which is the route of infection to the meninges. However, as all these episodes had otitis and meningitis, and the number of concomitant infection was low, we decided to describe them as one group. In conclusion, the findings from this large cohort of patients indicate that otogenic meningitis is a common form of meningitis associated with relatively low mortality, and may add useful information to current clinical practice.

## Supplementary Data

Supplementary materials are available at *Clinical Infectious Diseases* online. Consisting of data provided by the authors to benefit the reader, the posted materials are not copyedited and are the sole responsibility of the authors, so questions or comments should be addressed to the corresponding author.

## Supplementary Material

ciae221_Supplementary_Data

## Notes

***Author contributions.*** M. R.: substantial contribution to acquisition of data, analysis and interpretation of data, drafting/revising the article, final approval; T. M. v. S.: substantial contribution to acquisition of data, analysis and interpretation of data, revising the article, final approval; E. F. H.: revising the article, final approval; P. C.: revising the article, final approval; A. C.: revising the article, final approval; M. C. B.: substantial contribution to conception and design of the study, revising the article, final approval; D. v. d. B.: substantial contribution to conception and design of the study, revising the article, final approval.

***Acknowledgments.*** The authors thank all participating physicians in the Netherlands for their cooperation.

***Financial support.*** This work was supported by grants from the European Research Council (ERC Consolidator grant number 101001237 to M. B.), and the Netherlands Organization for Health Research and Development (Vici grant number 918.19.627 to D. v. d. B., Vidi grant number 917.17.308 to M. B.).
