# Supplementary material for: Otitis in Patients With Community-Acquired Bacterial Meningitis: A Nationwide Prospective Cohort Study
Source: Clin Infect Dis. 2024 Apr 24;79(2):329–35. doi: 10.1093/cid/ciae221 (PMC11327785; doi:10.1093/cid/ciae221)
Supplement: ciae221_Supplementary_Data [file ciae221_supplementary_data.docx]

**Table 1**: Surgery based on type of hospital where the patient was admitted^a^

|  | Academic hospitals  n = 95 | Large non-academic teaching hospitals  n = 316 | Small non-academic hospitals  n = 220 |
| --- | --- | --- | --- |
| ENT consultancy | 83/95 (87%) | 254/316 (80%) | 182/220 (83%) |
| Ear surgery^b^ | 50/83 (60%) | 130/254 (51%) | 107/182 (59%) |
| Myringotomy alone | 13/50 (26%) | 40/130 (31%) | 28/107 (26%) |
| Myringotomy with ventilation tube insertion | 19/50 (38%) | 70/130 (54%) | 48/107 (45%) |
| Mastoidectomy | 30/50 (60%) | 33/130 (25%)*** | 40/107 (37%)* |

Academic hospitals group was compared with each of the other two groups, singularly.

* p-value < 0.05 and >0.001, *** p-value<0.001.

^a^ 1 data missing on hospital admitted in 1 episodes out of 632

^b^ 27 episodes underwent both a myringotomy with ventilation tube insertion and mastoidectomy, 7 episodes underwent both a myringotomy alone and mastoidectomy

**Table 2:** Factors associated with unfavorable outcome in episodes with otogenic meningitis^a^

| Characteristics | Favorable outcome (n=442) | Unfavorable outcome (n=190) | Univariable odds ratio for unfavorable outcome (95% CI) | Multivariable odds ratio for unfavorable outcome (95% CI) | p value of multivariable analysis |
| --- | --- | --- | --- | --- | --- |
| Surgical Treatment |  |  |  |  |  |
| No surgery | 239/442 (54%) | 106/190 (56%) | Reference | Reference | - |
| Myringotomy alone | 55/442 (12%) | 19/190 (10%) | 0.78 (0.43-1.36) | 0.77 (0.42-1.37) | 0.38 |
| Myringotomy with ventilation tube insertion | 85/442 (19%) | 25/190 (13%) | 0.66 (0.40-1.08) | 0.64 (0.38-1.07) | 0.10 |
| Mastoidectomy | 63/442 (14%) | 40/190 (21%) | 1.43 (0.90-2.26) | 1.46 (0.90-2.34) | 0.12 |
| Age (years) |  |  |  |  |  |
| 16-29 | 13/442 (3%) | 4/190 (2%) | 0.81 (0.23-2.35) | 0.83 (0.22-2.51) | 0.76 |
| 30-64 | 296/442 (67%) | 112/190 (59%) | Reference | Reference | - |
| >65 | 133/442 (30%) | 74/190 (39%) | 1.47 (1.03-2.10) | 1.47 (1.01-2.13) | **0.04** |
| Male sex | 212/442 (48%) | 95/190 (50%) | 0.92 (0.66-1.30) | 0.95 (0.67-1.36) | 0.80 |
| GCS on admission^b^ | 11 (9-14) | 10 (7-12) | 0.85 (0.80-0.90) | 0.85 (0.80-0.91) | **<0.001** |
| *Streptococcus pneumoniae* | 389/442 (88%) | 170/190 (89%) | 1.16 (0.68-2.04) | 0.90 (0.51-1.64) | 0.72 |

Data are median (IQR) or n/N (%), unless stated otherwise. GCS= Glasgow Coma Scale.

^a^ Otogenic meningitis episodes with data available about ENT surgery (632 episodes). ^b^ Odds ratio is for a one point increase.

**Table 3**: Factors associated with unfavorable outcome in episodes with community-acquired bacterial meningitis

| Characteristics | Favorable outcome (n=1554) | Unfavorable outcome (n=979) | Univariable odds ratio for unfavorable outcome (95% CI) | Multivariable odds ratio for unfavorable outcome (95% CI) | p value of multivariable analysis |
| --- | --- | --- | --- | --- | --- |
| Age, years^a^ | 58 (43-67) | 66 (56-75) | 1.04 (1.03-1.05) | 1.03 (1.02-1.04) | **<0.001** |
| Sex, male | 771/1554 (50%) | 514/979 (53%) | 1.12 (0.96-1.32) | 1.22 (1.00-1.42) | **0.046** |
| Pretreatment with antibiotics | 164/1509 (11%) | 79/941 (8%) | 0.76 (0.58-1.00) | 0.83 (0.60-1.15) | 0.27 |
| Symptoms <24 h | 764/1516 (50%) | 388/909 (43%) | 0.74 (0.63-0.87) | 0.73 (0.60-0.89) | **0.002** |
| Immunocompromised* | 383/1521 (25%) | 373/948 (39%) | 1.92 (1.61-2.28) | 1.35 (1.10-1.66) | **0.005** |
| Otitis | 486/1554 (31%) | 210/979 (21%) | 0.60 (0.50-0.72) | 0.78 (0.62-0.98) | **0.033** |
| Pneumonia | 104/1512 (7%) | 130/906 (14%) | 2.53 (1.95-3.29) | 1.19 (0.87-1.65) | 0.28 |
| Headache | 1217/1434 (85%) | 509/722 (70%) | 0.39 (0.32-0.47) | 0.73 (0.57-0.94) | **0.014** |
| Nausea | 831/1339 (62%) | 366/714 (51%) | 0.63 (0.53-0.74) | 0.96 (0.77-1.18) | 0.68 |
| Rash | 149/1405 (11%) | 52/851 (6%) | 0.58 (0.43-0.79) | 0.78 (0.51-1.19) | 0.24 |
| Neck stiffness | 1088/1438 (76%) | 582/860 (68%) | 0.68 (0.57-0.81) | 0.74 (0.59-0.93) | **0.009** |
| Cranial nerve palsy | 73/1375 (5%) | 96/767 (13%) | 2.44 (1.84-3.24) | 2.29 (1.61-3.26) | **<0.001** |
| Focal neurologic deficit | 262/1440 (18%) | 230/805 (29%) | 1.77 (1.47-2.13) | 1.34 (1.05-1.70) | **0.018** |
| Heart rate (beats per min)^b^ | 96 (81-109) | 103 (88-120) | 1.20 (1.16-1.24) | 1.15 (1.10-1.20) | **<0.001** |
| Fever > 38°C | 1134/1516 (75%) | 645/926 (70%) | 0.77 (0.45-0.92) | 0.71 (0.57-0.90) | **0.004** |
| GCS on admission^c^ | 12 (10-14) | 10 (8-13) | 0.85 (0.83-0.87) | 0.88 (0.85-0.91) | **<0.001** |

| Characteristics | Favorable outcome (n=1554) | Unfavorable outcome (n=979) | Univariable odds ratio for unfavorable outcome (95% CI) | Multivariable odds ratio for unfavorable outcome (95% CI) | p value of multivariable analysis |
| --- | --- | --- | --- | --- | --- |
| *Streptococcus pneumoniae* | 1036/1554 (67%) | 719/979 (73%) | 1.39 (1.17-1.66) | 0.76 (0.60-0.97) | **0.025** |
| Positive blood culture | 980/1330 (74%) | 674/829 (81%) | 1.67 (1.38-2.04) | 1.14 (0.89-1.48) | 0.30 |
| C-reactive protein (mg/L) | 154 (68-260) | 234 (120-348) |  |  |  |
| <50 | 281/1507 (19%) | 92/926 (10%) | Reference | Reference |  |
| 50-150 | 457/1507 (30%) | 202/926 (22%) | 1.32 (1.00-1.76) | 1.26 (0.91-1.75) | 0.16 |
| >150 | 769/1507 (51%) | 632/926 (68%) | 2.51 (1.95-3.24) | 1.81 (1.33-2.48) | **<0.001** |
| Thrombocyte count | 207 (159-258) | 180 (129-245) |  |  |  |
| <150 | 314/1478 (21%) | 300/889 (34%) | 1.97 (1.64-2.36) | 1.25 (1.00-1.55) | 0.050 |
| 150-450 | 1134/1478 (77%) | 568/889 (64%) | Reference | Reference |  |
| >450 | 30/1478 (2%) | 21/889 (2%) | 1.38 (0.79-2.36) | 1.01 (0.52-1.98) | 0.97 |
| CSF protein (g/L) | 3.5 (2.0-5.8) | 4.5 (2.6-6.8) |  |  |  |
| <0.5 | 36/1463 (2%) | 19/903 (2%) | Reference | Reference |  |
| 0.5-1.5 | 206/1463 (14%) | 76/903 (8%) | 0.67 (0.37-1.24) | 0.77 (0.36-1.63) | 0.49 |
| >1.50 | 1221/1463 (83%) | 808/903 (89%) | 1.22 (0.71-2.16) | 0.85 (0.41-1.79) | 0.67 |
| Cerebrospinal fluid white cell count (cell per µL) | 3060 (1020-7645) | 1252 (215-4829) |  |  |  |
| <100 | 90/1481 (6%) | 157/920 (17%) | 3.90 (2.96-5.15) | 2.64 (1.83-3.80) | **<0.001** |
| 100-999 | 273/1481 (18%) | 271/920 (29%) | 2.23 (1.83-2.73) | 2.03 (1.59-2.58) | **<0.001** |
| 1000-10000 | 855/1481 (58%) | 389/920 (42%) | Reference | Reference |  |
| >10000 | 263/1481 (18%) | 103/920 (11%) | 0.86 (0.67-1.10) | 0.79 (0.59-1.06) | 0.11 |
| CSF:blood glucose ratio | 0.1 (0.01-0.31) | 0.02 (0.01-0.17) |  |  |  |
| <0.25 | 922/1414 (65%) | 664/824 (81%) | 1.83 (1.29-2.64) | 2.30 (1.34-3.96) | **0.003** |
| Characteristics | Favorable outcome (n=1554) | Unfavorable outcome (n=979) | Univariable odds ratio for unfavorable outcome (95% CI) | Multivariable odds ratio for unfavorable outcome (95% CI) | p value of multivariable analysis |
| 0.25-0.5 | 394/1414 (28%) | 126/824 (15%) | 0.85 (0.58-1.26) | 1.24 (0.71-2.15) | 0.44 |
| >0.5 | 98/1414 (7%) | 34/824 (4%) | Reference | Reference |  |

The study included 2533 out of 2548 episodes of community-acquired meningitis; 15 episodes did not have data on outcome. Data are median (IQR) or n/N (%), unless stated otherwise. The multivariable analysis used an imputed dataset with 60 imputation rounds, all variables in the table were entered in the multivariable logistic regression model simultaneously. CSF=cerebrospinal fluid; GCS= Glasgow Coma Scale. *Patient was defined immunocompromised if either one of the following conditions were present: active cancer, immunosuppressive therapy, history of alcohol abuse, diabetes mellitus, splenectomy, HIV infection.

^a^ Evaluated in 2533 episodes; ^b^ Evaluated in 2395 episodes; odds ratio is for an increase of ten beats per min; ^c^ Evaluated in 2490 episodes; odds ratio is for a one point increase.
